# Hair Cell Protection from Ototoxic Drugs

**DOI:** 10.1155/2021/4909237

**Published:** 2021-07-11

**Authors:** Peng Wu, Xianmin Wu, Chunhong Zhang, Xiaoyun Chen, Yideng Huang, He Li

**Affiliations:** Department of Otolaryngology, First Affiliated Hospital of Wenzhou Medical University, Wenzhou City, 325000 Zhejiang Province, China

## Abstract

Hearing loss is often caused by death of sensory hair cells (HCs) in the inner ear. HCs are vulnerable to some ototoxic drugs, such as aminoglycosides(AGs) and the cisplatin.The most predominant form of drug-induced cell death is apoptosis. Many efforts have been made to protect HCs from cell death after ototoxic drug exposure. These mechanisms and potential targets of HCs protection will be discussed in this review.And we also propose further investigation in the field of HCs necrosis and regeneration, as well as future clinical utilization.

## 1. Introduction

Hearing loss is the most common sensory impairment in humans. It is estimated that there were 466 million people living with hearing loss in 2018 [1]. Hearing loss is often caused by death of sensory hair cells (HCs) in the inner ear, which function in transducing the sound waves into electric signals [2–6]. HCs are vulnerable to a variety of different stresses, such as aging, acoustic trauma, genetic disorders, infection, and exposure to some ototoxic drugs [7–13]. Unfortunately, the mammals only have very limited HC regeneration ability, and the death of HCs in mammals is irreversible, thus leading to permanent hearing deficit [13–18]. Although hearing loss is not a life-threatening disease, it can affect the patient's quality of life, especially in children, which will cause delays in language acquisition and dumb. That will cause significant burden on families and society.

Currently, the most effective and convenient protection is avoiding exposure to known ototoxic drugs. Although there are several drugs that can injure HCs, the most commonly encountered ototoxic drugs are the aminoglycosides (AGs) and the antineoplastic agent cisplatin. AGs are the most commonly prescribed antibiotics, such as gentamicin, amikacin, kanamycin, and neomycin, which are usually used in the treatment of infections caused by aerobic gram-negative bacteria. Cisplatin is a platinum-based chemotherapeutic drug, which is often used for the chemotherapy of malignant tumors. But the ototoxicity limits the clinical application of these two kinds of drugs. Both AGs and cisplatin can induce apoptotic cell death in HCs, especially the outer HCs of the basal turn [9, 19–23].

HCs can undergo cell death through apoptosis and necrosis. But the most predominant form of drug-induced cell death is apoptosis. In order to protect HCs from ototoxic insult, a better understanding of the mechanisms of aminoglycoside- and cisplatin-induced hair cell death is required. Current studies of these apoptotic cell death mechanisms and potential targets of HC protection are discussed in this review.

## 2. Mechanism and Protection

### 2.1. Route of Ototoxic Drugs into Hair Cells

After systemic administration, ototoxic drugs can pass the blood-labyrinth barrier (BLB) and enter the endolymph via the Reissner's membrane, especially via the stria vascularis [24]. After that, they enter into HCs and cause cell death.

Multiple pathways for entry of AGs and cisplatin into HCs exist. One pathway is endocytosis at the apical and synaptic poles of HCs, although direct evidence for its involvement in cytotoxicity has not been found [25, 26]. Transport through ion channels, especially mechanoelectrical transducer (MET) channel, is supposed to play an important role in AGs uptake into HCs [25–28]. Some researchers suggest AGs and cisplatin can enter the HCs through MET channel [25, 29, 30] or Copper Transporter 1 (CTR1) [31], respectively, which are located at the top of hair cell stereocilia. Other studies suggest that MET channel is also a major contributor to the entry of cisplatin into HCs, at least in the zebrafish [32, 33]. But a direct interaction between cisplatin and mammalian MET channels has not been reported. Some researchers reveal that cisplatin entry into cochlear and HCs is also mediated by organic cation transporter (OCT), and the expression of OCT2, an isoforms of OCT, has been detected in HCs, as well as in stria vascularis [31, 34]. There is also evidence for the participation of transient receptor potential (TRP) channels, a family of polymodal ion channels activated by a variety of physical and chemical stimulation, such as oxidative stress, tissue damage, and inflammation [28]. TRP channels, such as TRPA1, TRPV1, and TRPV4, are additional candidate aminoglycoside-permeant channels, and all of them are found expressed in the HCs [35–37]. Exposure to immunostimulatory lipopolysaccharides, to simulate of bacterial infections, increased the cochlear expression of TRPV1 and hair cell uptake of gentamicin, thus, exacerbate ototoxicity of AGs [38]. In murine cochlear cultures, when the MET channels were disabled, the activated TRPA1 channels will facilitate the uptake of gentamicin [37].

### 2.2. Efforts in Inhibiting the Uptake of Drugs

Avoiding ototoxic drugs entry into HCs is the primary step. On the level of the MET channel, there are two possibilities exist. The first one is steric modification of the chemical structure of drugs. The MET channel pore, which has a diameter at its narrowest part of at least 1.25-1.5 nm, is large enough to allow AGs to enter the hair cell cytosol [39]. Therefore, widening the AG diameter by binding of certain molecules appears a promising strategy to inhibit AGs passing through the MET channel. But this biding must be irrelevant for antimicrobial activity [40]. The second way is blocking the MET channel to prevent ototoxic drugs entering HCs, especially for AGs. MET channel blocker, such as ORC-13661, can protect HCs against both AGs and cisplatin [41]. Because blocking of the MET channel would prevent hair cell depolarization and affect hearing function, therefore, the blockage must be temporary [42, 43].

Myosin7a is supposed to mediate AG endocytosis, and the uptake of AGs was decreased in Myosin7a mutant mice. This indicate a promising target for HC protection [44].

Intratympanic administration of copper sulfate, a CTR1 inhibitor, or knockdown of CTR1 with small interfering RNA can decrease the uptake and cytotoxicity of cisplatin and prevent hearing loss caused by cisplatin, both in vitro and in vivo [31]. OCT knockout or inhibition of OCT with cimetidine protects HCs against cisplatin-induced ototoxicity [34].

## 3. The Involvement of Mitochondrial Dysfunction and DNA Damage

The entry of AGs into HCs can lead to mtDNA mutations and thus affect the RNA translation and protein synthesis within mitochondria [45] and therefore leading to a decrease in ATP synthesis. With the decrease of energy production, the mitochondrial membrane integrity is compromised and thus leading to the leakage of cytochrome c, the generation of reactive oxygen species (ROS), and activation of stress kinases [46, 47]. The accumulation of ROS and cytochrome c will lead to the activation of the upstream caspases and subsequent apoptotic cell death. On the other hand, both ROS and stress kinases can cause cell death directly, as well as by amplifying insults targeting the mitochondria. And ROS can also cause mtDNA defects.

As for cisplatin, the ototoxic mechanism has been shown to be associated with several factors, such as oxidative stress, DNA damage, and inflammatory cytokines. Several studies have implicated the mitochondrial pathways in the apoptosis of HCs after cisplatin administration [48]. Exposure to cisplatin can also cause excessive generation of ROS via the NADPH-oxidase (NOX) pathway [49, 50], which will activate the mitochondrial apoptosis pathway that mentioned above. The signal transducers and activators of transcription 1 (STAT1) is an important mediator of cell death, and the STAT1 phosphorylation was found in HCs after exposure to cisplatin. STAT1 is involved in the response to the release of ROS, inflammatory cytokines, and DNA damage [51].

All these mechanisms of drug-induced hair cell death and protection will be discussed below.

### 3.1. Reactive Oxygen Species

ROS are mainly generated by the mitochondria in mammalian cells. AGs can combine with iron salts, and the iron-AG complexes catalyze free radical reactions and lead to ROS generation [52]. As mentioned above, AGs decrease the ATP synthesis, which will increase the permeability of mitochondrial transmembrane and the leakage of cyt-c and ROS. The ROS can also generate via the NOX3 pathway after cisplatin exposure. The ROS overload leads to the depletion of the cochlear antioxidant enzyme system (e.g., superoxide dismutase, catalase, glutathione peroxidase, and glutathione reductase), which scavenges and neutralizes the generated superoxide and hydrogen peroxide [53]. The release of ROS causes further damage to mitochondrial components, such as mtDNA, mitochondrial membranes, and respiratory chain proteins, as well as nuclear DNA associated with mitochondrial function [54]. The ultimate effect of increased ROS generation is to promote apoptotic cell death, as described above.

### 3.2. Neutralization of Reactive Oxygen Species

Some studies have reported that antioxidants can promote HC survival in drug-induced ototoxicity, including coenzyme Q10 [55]; *α*-lipoic acid [56]; D-methionine [57]; thiourea [58]; vitamins B, C, and E [59]; N-acetylcysteine (NAC) [60]; and hormone melatonin [61]. Knockdown of NOX3 by intratympanic delivery of short interfering RNA (siRNA) protects against cisplatin-induced HC death [62]. Reducing the expression of TRPV1 or NOX3 can inhibit the ROS generation and the transcription factor STAT1 activation. And STAT1 activation will promote proapoptotic actions of cisplatin [63]. This indicates the inhibition of TRPV1 or NOX3 as promising approaches for reducing cisplatin ototoxicity. Another candidate strategy is the use of iron chelators, 2,3-dihydroxybenzoate [64], and acetylsalicylate (ASA) [65], which can compete with AGs for iron binding.

However, effects of these long-term treatments remain to be studied.

### 3.3. Caspase-Mediated Apoptosis

It has generally been accepted that the ototoxic drug-induced hair cell death shares a common pathway: caspase activation.

Caspases are divided into upstream and downstream members, which are normally inactive by binding with inhibitor of apoptosis proteins (IAP) [66, 67]. The upstream caspases are activated by proapoptotic signals, such as cytochrome c [68, 69], p53 [49], antiapoptotic Bcl-2 proteins [70, 71], tumor necrosis factor (TNF) family [72], and nuclear factor kappa B (NF-*κ*B) [73]. And the downstream caspases are activated by upstream caspases.

Caspase-8 is an upstream member, which is linked to membrane-associated death receptors. Caspase-8 can activate by ligands such as Fas or TNF-*α* and subsequently activate downstream caspases such as caspases-3, -6, and -7 [72, 74]. Although caspase-8 is activated in HCs after AG administration [75], inhibition of this pathway does not prevent HC death or prevent caspase-3 activation [76]. Thus, it does not play a key role in HC death.

Caspase-9 is also an upstream member, which is triggered by nonreceptor stimulation, such as cytokine c releasing from mitochondrial [69]. After activation, caspase-9 can cleave and activate downstream caspases-3, which eventually leading to apoptotic HC death [75]. Caspase-3 is a downstream member, which mediates apoptotic program by cleaving proteins necessary for cell survival, such as cytoskeletal proteins [77]. The cisplatin-induced activation of caspase-9 and caspase-3 was seen in HEI/OC1 cells [78] and UB/OC1 cells [79].

### 3.4. Inhibition of Caspase Members

Studies have shown that intracochlear administration with specific inhibitors of caspase-9 or caspase-3 can prevent AG-induced or cisplatin-induced HC death and hearing loss [48, 80]. Caspase inhibitors, such as z-VAD-FMK and z-LEHD-FMK, can protect HCs against AG-induced cell death [81, 82]. Intracochlear perfusions with caspase-3 inhibitor (z-DEVD-fmk) and caspase-9 inhibitor (z-LEHD-fmk) prevent hearing loss and loss of HCs in cisplatin treated guinea pigs [48]. Several other efforts targeting the different steps in caspase activation are also promising. For example, NF-*κ*B inhibitors, such as Bay 11-7085 or SN-50, can inhibit cisplatin-induced caspase-3 activation and apoptosis in HEI/OC1 cells [78].

### 3.5. BCL-2 Family

The Bcl-2 family can be categorized as antiapoptotic (e.g., Bcl-2 and Bcl-XL) or proapoptotic (e.g., Bax, Bak, Bcl-Xs, Bid, Bad, and Bim) members [83, 84]. Antiapoptotic Bcl-2 members can bind to proapoptotic Bcl-2 members, which will neutralize the proapoptotic signal [85]. The balance between the antiapoptotic and proapoptotic members is crucial for the living of the cell. When the balance tilts to proapoptosis, the proapoptotic Bcl-2 members, such as Bax and Bid, will translocate from the cytoplasm to the mitochondria, which will increase the permeability of mitochondrial transmembrane and lead to the generation of ROS and leakage of cytochrome c into the cytoplasm, thus eventually activate caspase-9 and caspase-3 and lead to apoptotic cell death as mentioned above [86, 87]. Recently, the increased expression of Bax and the decreased expression of Bcl-XL were observed in UB/OC-1 cells after cisplatin treatment [79]. The overexpression of Bcl-2 can inhibit the release of cytochrome c, thereby inhibiting the apoptosis cascade. This has been confirmed by some researchers in cochlear cell line or mouse utricles following AGs or cisplatin exposure [48, 70, 88].

### 3.6. Efforts on Targeting the Bcl-2 Family

Targeting the Bcl-2 family as the upstream mediator of apoptosis can prevent AG-induced hair cell death. Some studies reveal that overexpression of the antiapoptotic Bcl-2 members can inhibit apoptotic hair cell death following AG exposure *in vitro* and *in vivo* [70, 87, 89], while epigallocatechin gallate (EGCG), a known inhibitor of STAT1, can reverse the balance of Bax and Bcl-XL to antiapoptotic, which will protect HCs against apoptosis after cisplatin administration [79].

### 3.7. The c-jun NH2-Terminal Kinases (JNKs)

The c-jun NH2-terminal kinases (JNKs) are key modulators of apoptosis, which are activated in response to cellular insults, such as generation of ROS, in HCs treated with neomycin and cisplatin [90, 91]. JNK activation acts as upstream of cytochrome c redistribution and caspase activation [92, 93]. When activated, JNKs can activate the transcription factors c-Jun, c-FOS, ELK-1, and Bcl-2. After AG administration, the increased JNKs, c-Jun, c-FOS, and Bcl-2 have been observed in HCs [94–96].

### 3.8. Inhibitors of the JNK Pathway

JNK inhibitors such as CEP-1347 [97] and CEP 11004 [91] can attenuate hair cell loss following AG administration. But, JNK inhibitor does not protect HCs against cisplatin-induced cell death, nor does it prevent redistribution of cytochrome c [48].

**Figure 1:**
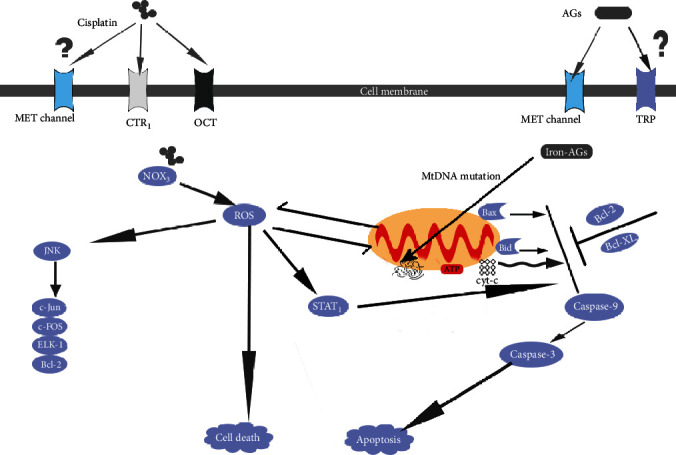
AGs and cisplatin enter the HCs through MET channel or CTR1. The iron-AG complexes cause mtDNA mutations and affect the protein synthesis. The decrease of ATP synthesis, as well as the translocation of proapoptotic Bcl-2 members (Bax and Bid) will increase the permeability of mitochondrial transmembrane. Thus, leading to the leakage of cyt-c and ROS. The cyt-c will lead to the caspase activation and apoptosis. ROS can cause cell death or amplify insults targeting mitochondria. The iron-AG complexes can catalyze free radical reactions and lead to ROS generation. ROS can also generate via the NOX3 pathway. When JNK was activated by ROS, it will activate some key modulators of apoptosis (c-Jun, c-FOS, ELK-1, and Bcl-2). ROS can also activate STAT1, which will promote proapoptotic actions of cisplatin. AG: aminoglycoside; MET: mechanoelectrical transducer; CTR1: Copper Transporter 1; OCT: organic cation transporter; TRP: transient receptor potential; NOX3: NADPH-oxidase 3; ROS: reactive oxygen species; cyt-c: cytochrome c; STAT1: transcription factor; JNK: c-jun NH2-terminal kinase.

The mechanisms of AG-induced and cisplatin-induced HCs death are summarized in Figure 1.

### 3.9. Other Promising Targets

There are some other mechanisms underlying the ototoxic of AGs and cisplatin, such as heat shock proteins (HSP), p53, and NF-*κβ* as well as calcium-dependent proteases, and so on. Researchers have achieved promising outcomes. For example, overexpression of HSP-70 in transgenic mice can protect HCs against both aminoglycoside- and cisplatin-induced hair cell death [98, 99]. It is indicated that p53 acts upstream of mitochondrial apoptotic pathway and downregulation of the p53 gene protects HCs from cisplatin-induced Bax translocation, caspase-3 activation, cytochrome c translocation, and cell death [100]. Although p53 inhibitor protects against cisplatin-induced ototoxicity, the systemic application will interfere with the anticancer efficacy of cisplatin, while it is revealed that the intratympanic application of p53 inhibitor, such as pifithrin-a, protects auditory function without compromising the anticancer efficacy of cisplatin [100]. It has been revealed that Wnt/*β*-catenin signaling has an important role in protecting HCs against neomycin-induced HC loss. The overexpression of *β*-catenin can reduce forkhead box O3 transcription factor (Foxo3) and Bim expression and ROS levels after neomycin exposure [11]. This might be a new therapeutic target. Some researchers used rapamycin, an autophagy activator, to increase the autophagy activity and found that the ROS levels, apoptosis, and cell death were significantly decreased after neomycin or gentamicin exposure, suggesting that autophagy might be correlated with AG-induced HC death [101]. It is also revealed that meclofenamic acid can attenuate cisplatin-induced oxidative stress and apoptosis in HEI-OC1 cells, by inhibiting cisplatin-induced upregulation of autophagy [12].

### 3.10. Potential Drug Targets

With increased understanding of ototoxic cell death, a numerous of therapeutic efforts have been made to target different steps of in HC death. The HEI-OC1 and UB/OC-1 cell lines, organ explants, larval zebrafish lateral-line neuromasts, and some animal model (e.g., chicken, rat, mouse, and guinea pig) are the most commonly used research strategies. The delivery of test compounds can be performed by intratympanic, intraperitoneal, intramuscular, subcutaneous, intracochlear, and oral administration. Potential drug targets for treatment of AG and cisplatin ototoxicity are summarized in Table 1.

## 4. Conclusion

As discussed above, many efforts have been made to protect HCs from cell death after ototoxic drug exposure. The outcomes are promising, but risks also arise. For example, endotoxemia-mediated inflammation can enhance aminoglycoside trafficking across the BLB and potentiate AG-induced ototoxicity. This indicates that patients with severe infections are at greater risk of AG-induced hearing loss than previously recognized. Systemic interference with cell signaling pathways may also have unknown physiological consequences. So, it is extremely different to apply clinically. For example, as an iron chelator, ASA itself is ototoxic and can cause tinnitus, vertigo, and hearing loss. On the other side, long-term treatment with antiapoptotic drugs bears a potential carcinogenic risk, as apoptosis is crucial in preventing uncontrolled cell proliferation. Although antioxidants are well established as otoprotectants, some studies show that administered of a single antioxidant in high oxidative environment would be rapidly oxidized and produce only transient benefit in preventing hearing loss [102]. As for AGs can remain in HCs for months, thus, use of a single antioxidant in high-risk human populations has not produced expected benefits; the outcomes of long-term and mixture administration with other drugs are also need to be well studied.

A variety kind of insults to the inner ear can cause HC death and hearing loss. Although the most predominant form of drug-induced cell death is apoptosis, necrotic features are also seen in HCs following AG exposure [19]. This suggests that the apoptotic and necrotic cell death that occurs in HCs may share among many ototoxic events, while the necrosis and associated pathways are still unclear in HCs after ototoxic drug exposure. Research in the mechanisms of regulated necrosis in HCs may improve our understanding of the complex communications between different signaling cascades. On the other side, great progresses have been made in the field of HC regeneration. For example, it has been reported that Lgr5-expressing cells can differentiate into HCs [17], and several genes have been identified that regulate the regeneration of HCs [13, 18]. These are also promising strategies.

Thus, a full understanding of the mechanisms in ototoxic drug-induced hearing loss still remains urgent, and the possibility of future clinical utilization is also need to be well evaluated.

## Figures and Tables

**Table 1:** Potential drug targets for treatment of AG and cisplatin ototoxicity.

Compound	Ototoxic drug	Mechanism	Materials and methods	References
ORC-13661	AG and cisplatin	Block MET channel	Mouse cochlear cultures, *in vitro* zebrafish, *in vitro*	[41]
Copper sulfate	Cisplatin	CTR1 inhibitor, inhibit uptake	HEI-OC1 cells, *in vitro* Mice, *in vivo*, i.t.	[31]
Cimetidine	Cisplatin	OCT blocker, inhibit uptake	Mice, *in vivo*, i.p.	[34]
Coenzyme Q10	Cisplatin	Antioxidant	Rat, *in vivo*, oral administrations	[55]
*α*-Lipoic acid	AG	Antioxidant	Guinea pigs, *in vivo*, i.m.	[56]
D-Methionine	AG	Antioxidant	Guinea pigs, *in vivo*, i.p.	[57]
Thiourea	Cisplatin	Antioxidant	Guinea pigs, *in vivo*, intracochlear perfusion by osmotic pump	[58]
Vitamins B, C, and E	Cisplatin	Antioxidant	Rat, *in vivo*, i.p.	[59]
N-Acetylcysteine	AG	Antioxidant	Rat, *in vivo*, i.p.	[60]
Hormone melatonin	Cisplatin	Antioxidant	Rat, *in vivo*, i.p.	[61]
siRNA	Cisplatin	Inhibit TRPV1 or NOX3 Inhibit ROS generation and STAT1 activation	UB/OC-1 cells, *in vitro* Rat, *in vivo*, i.t.	[62]
2,3-Dihydroxybenzoate	AG	Iron chelators Compete with AG for iron binding	Guinea pigs, *in vivo*, i.p.	[64]
Acetylsalicylate	AG	Iron chelators, compete with AG for iron binding Antioxidant	Guinea pigs, *in vivo*, oral administration	[65]
EGCG	Cisplatin	STAT1 inhibitor Antiapoptotic	Rat, *in vivo*, oral administrations	[79]
Bay 11-7085	Cisplatin	NF-*κ*B inhibitors Inhibit caspase-3 activation	HEI/OC1 cells, *in vivo*	[78]
SN-50	Cisplatin	NF-*κ*B inhibitors Inhibit caspase-3 activation	HEI/OC1 cells, *in vivo*	[78]
z-VAD-FMK	AG	General caspase inhibitor	Guinea pigs, *in vivo*, intracochlear perfusion by osmotic pump	[81]
z-LEHD-FMK	AG	Caspase-9 inhibitor	Guinea pigs, *in vivo*, intracochlear perfusion by osmotic pump	[81]
z-DEVD-fmk	Cisplatin	Caspase-3 inhibitor	Guinea pigs, *in vivo*, intracochlear perfusion by minipump	[48]
z-LEHD-fmk	Cisplatin	Caspase-9 inhibitor	Guinea pigs, *in vivo*, intracochlear perfusion by minipump	[48]
CEP-1347	AG	JNK inhibitor	Guinea pigs, *in vivo*, s.c.	[97]
CEP 11004	AG	JNK inhibitor	Chicken vestibular hair cell culture, *in vitro*	[91]
Pifithrin-a	Cisplatin	p53 inhibitor Inhibit mitochondrial apoptotic pathway	Mouse cochlear culture, *in vitro*	[100]

AG: aminoglycoside; MET: mechanoelectrical transducer; CTR1: Copper Transporter 1; OCT: organic cation transporter; siRNA: short interfering RNA; TRP: transient receptor potential; NOX3: NADPH-oxidase 3; ROS: reactive oxygen species; STAT1: transcription factor; EGCG: epigallocatechin gallate; JNK: c-jun NH2-terminal kinase; i.t.: intratympanic; i.p.: intraperitoneal; i.m.: intramuscular; s.c.: subcutaneous.
